# Exercise and Nutrition for Healthy AgeiNg (ENHANce) project – effects and mechanisms of action of combined anabolic interventions to improve physical functioning in sarcopenic older adults: study protocol of a triple blinded, randomized controlled trial

**DOI:** 10.1186/s12877-020-01900-5

**Published:** 2020-12-10

**Authors:** Lenore Dedeyne, Jolan Dupont, Katrien Koppo, Sabine Verschueren, Jos Tournoy, Evelien Gielen

**Affiliations:** 1 Department of Public Health and Primary Care, KU Leuven, Leuven, Belgium; 2 Department of Geriatric medicine, UZ Leuven, Leuven, Belgium; 3 Department of Movement Sciences, KU Leuven, Leuven, Belgium; 4 Department of Rehabilitation Sciences, KU Leuven, Leuven, Belgium

**Keywords:** Sarcopenia, Frailty, Older adults, Community-dwelling, Randomized controlled trial, Compliance, Protein supplementation, Omega-3 fatty acids, Exercise intervention

## Abstract

**Background:**

The Exercise and Nutrition for Healthy AgeiNg (ENHANce) project aims to assess the combined effects of exercise and nutritional interventions to prevent loss of skeletal muscle mass and function with ageing, and to determine the underlying mechanisms of action.

**Methods:**

One hundred eightycommunity-dwelling sarcopenic individuals (≥ 65 years) are allocated in a randomized controlled trial (RCT) in a 1:1 ratio into five groups for a 12-week intervention period, followed by a 12-week follow-up period: 1) exercise intervention +protein placebo +omega-3 fatty acids placebo; 2) protein +omega-3 fatty acids placebo; 3) exercise intervention +protein +omega-3 fatty acids placebo; 4) exercise intervention +protein +omega-3 fatty acids; 5) protein placebo +omega-3 fatty acids placebo. All interventions are in line with recommendations of expert groups such as the American College of Sports Medicine and the PROT-AGE study group and individualized to the physical capabilities and nutritional intake of each participant. Sarcopenia is diagnosed by the assessment of gait speed, handgrip strength (Jamar handheld dynamometer), chair stand test and muscle mass (DXA) according to the European Working Group on Sarcopenia in Older People (EWGSOP2) criteria. Participants, researchers and statisticians are blinded to omega-3 fatty acids and protein treatment. Compliance to the exercise program, protein and omega-3 fatty acids interventions is objectively measured, by monitoring movement by an activity monitor, determining nitrogen content in urine and analyzing the fatty acid composition of the red blood cell membrane. The primary outcome of the RCT is the change in Short Physical Performance Battery (SPPB) score. Secondary endpoints are, among others, changes in muscle mass, strength and function, objective compliance to interventions, changes in muscle and blood biomarkers related to sarcopenia, cognition, quality of life and falls.

**Discussion:**

This RCT in well-defined sarcopenic older adults assesses the effects of combined anabolic interventions, including the additive effects of omega-3 fatty acids supplements, compared to single or placebo interventions. Compliance with the exercise intervention and with the intake of nutritional supplements is measured objectively. Also, blood and muscle samples will be used to explore the underlying determinants that contribute to the mechanism of action of anabolic interventions.

**Trial registration:**

Clinicaltrials.gov: NCT03649698, retrospectively registered at 28 August 2018, first participant was randomized 16 February 2018.

## Background

Sarcopenia, the age-related loss of muscle mass and muscle strength, is a key component of musculoskeletal frailty and predisposes older adults to disability, immobility, falls, fractures and death [1]. This progressive muscle disorder dramatically impacts an older person’s independence and quality of life (QoL) [1–3]. For example, persons with low muscle mass have a higher risk to become physically dependent compared to persons with normal mass, and this risk is even greater in persons with both low muscle mass and low muscle function [4]. Since sarcopenia is prevalent (varying from 9.9 to 40.4% depending on the definition of sarcopenia) in community-dwelling older adults [5] and the life expectancy continues to rise (27.8% of the European population will be over 65 years by 2050 [6]) the number of older adults with sarcopenia is expected to rise in the future [7].

Expert groups recommend physical exercise and nutritional interventions, or the combination, to prevent and treat sarcopenia [8]. However, results of previous randomized controlled trials (RCT’s) examining the combined effect of exercise and proteins on muscle mass and function are conflicting [9, 10]. This may be explained by a suboptimal exercise program (frequency, intensity, duration, type) and/or suboptimal protein supplement (dose, timing, amino acid content). Therefore, the interventions in the ENHANce study are in line with current recommendations as suggested by the American College of Sports Medicine [11] and the PROT-AGE study group [12]. Moreover, in previous RCTs, these interventions were not adapted or individualized to the subject’s physical capabilities and his/her nutritional status, although this is important to obtain maximal effects [3]. In addition, although there is growing interest in the use of omega-3 fatty acids supplementation in the treatment and prevention of sarcopenia [13–15] there is only limited data on the additional effect of omega-3 fatty acids combined with exercise and protein interventions on sarcopenia, with previous research using suboptimal protein and omega-3 fatty acids dosages [14]. Furthermore, in most of the trials combining physical exercise and nutritional interventions, objective compliance of the participants to the interventions was not monitored, implicating the interpretation of the results [8, 10, 16]. Lastly, the physiologic effects and underlying mechanisms of action of the combination of an exercise program and food compounds have been poorly investigated in human subjects and need further research [17, 18].

The overall objective of the ENHANce project is to assess the physical and biochemical effects and the mechanisms of action of combined individualized and optimized nutritional and exercise interventions in sarcopenic older people [19]. The primary aim is to examine effects on physical performance of an optimized and individualized combination of a home-based exercise program with protein supplementation compared to a home-based exercise program alone. The secondary aim is to assess in an exploratory way, the effects on other outcomes (including muscle mass, muscle strength and muscle function) of other interventions in a 1:1 ratio allocated 5-arm RCT (protein alone, exercise alone, protein and exercise, protein and exercise and omega-3 fatty acids, placebo interventions). The tertiary aim is to objectively measure the compliance with the anabolic interventions. The last aim is to explore the change in metabolic parameters and blood and muscle biomarkers in response to the anabolic interventions. We hypothesize that the optimized and individualized combination of a home-based exercise program and protein supplementation will improve physical performance more than a single physical exercise intervention. We also hypothesize that multi-domain interventions improve outcomes more than single domain interventions and that higher compliance will result in higher intervention effects.

## Methods

### General design

**Fig. 1:**
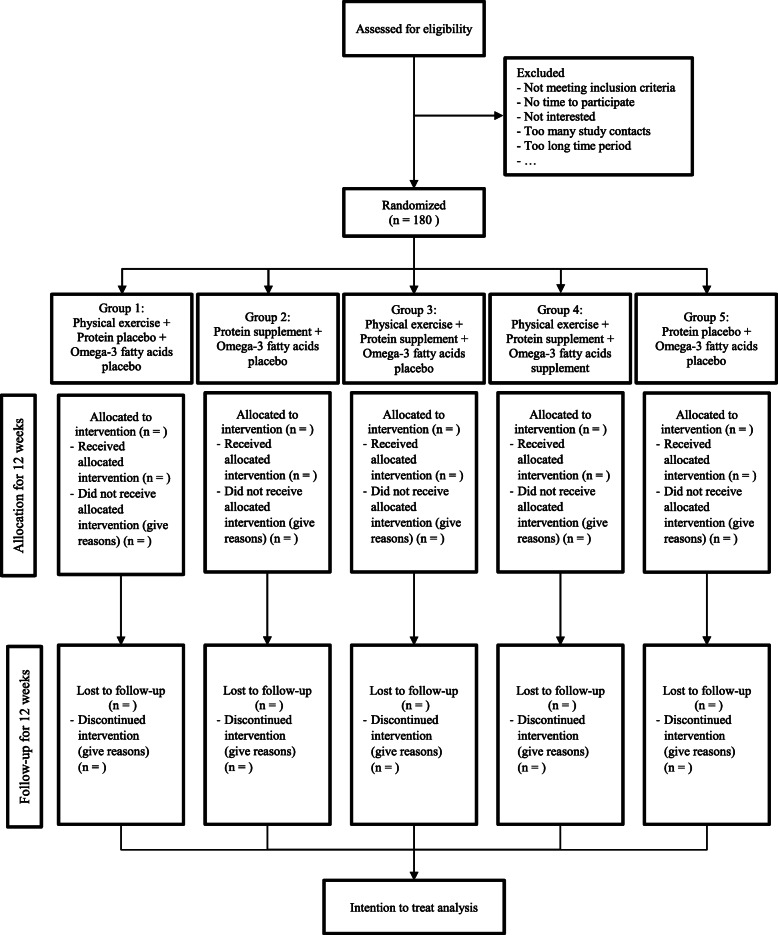
Study flow chart

The ENHANce project is a single center, parallel group, RCT, conducted in UZ Leuven, an academic hospital in Leuven, Belgium. Participants are randomly allocated in a 1:1 ratio into five arms (Fig. 1): group 1) ‘Ex’: Exercise intervention + protein placebo + omega-3 fatty acids placebo; group 2) ‘Prot’: Protein + omega-3 fatty acids placebo; group 3) ‘Ex + Prot’: Exercise intervention + protein + omega-3 fatty acids placebo; group 4) ‘Ex + Prot + omega-3 fatty acids’: Exercise intervention + protein + omega-3 fatty acids; group 5) ‘Ctr’: Control group: protein placebo + omega-3 fatty acids placebo. The 12-week intervention period is preceded by a preparation period of at least 28 days and is followed by a 12 week follow-up period without intervention.

### Participants

One hundred eighty sarcopenic people (> 65 years) will be included in the RCT. Participants are recruited in the local community of Leuven and surrounding cities in Flanders, Belgium. Older persons are contacted through information distributed by older person organizations, day care facilities in private and hospital setting, advertisements in local newspaper etc. Medical doctors are also contacted to recruit older persons. The participants must meet the following criteria: have probable or confirmed sarcopenia according to EWGSOP2 [3], able to communicate in Dutch, English or French, no allergy to milk, soy, peanut or peanut oil, Mini-Mental State Examination (MMSE) > 21 [20], no terminal illness with a prognosis less than 6 months, a protein intake lower than 1.5 g/kg body weight (BW)/day estimated in the week after screening visit, no participation in a training program more or equal than twice per week for the last 6 months, no uncontrolled disease or acute cardiovascular problems (or positive advise of doctor to perform physical activities), 25-hydroxyvitamin D blood concentration > 20 ng/L, glomerular filtration rate > 30 ml/min/1.73m^2^, fasting glycaemia < 126 mg/dl, no anti-diabetic medication, no impairments or diseases that impose problems to study participation according to the researchers.

#### Muscle biopsy substudy

In a substudy of the ENHANce RCT, a muscle biopsy of the *m. vastus lateralis* is taken in 40 participants (8 of each arm) at baseline and after the intervention period (week 12). Participants included in this substudy must fulfill the criteria as specified for the main study, and additional exclusion criteria are: under treatment with anticoagulants or oral corticosteroids medication and allergy to lidocaine.

### Study procedures

#### Study design

The ENHANce study starts with a screening visit to check eligibility, followed by a preparation period of approximately one month. During this preparation period all participants start the intake of vitamin D (800 International Unit (IU) cholecalciferol per day) and receive an omega-3 fatty acids supplement or omega-3 fatty acids placebo, to obtain steady state conditions before start of the intervention period. After the preparation period, the intervention period begins with a baseline visit and ends with a week-12 visit. The follow-up period starts immediately after the intervention period and ends after another 12-week period. During the screening visit, study procedures are explained and written informed consent is obtained by the study coordinator. Afterwards, fulfillment with the inclusion criteria is checked.

#### Assessment of inclusion criteria

Measures of body composition, handgrip strength, habitual gait speed and chair stand test are taken. Body weight (SECA, model no 8801321009, SECA UK Ltd.) to the nearest 0.1 kg and height (Harpenden stadiometer, Holtain Ltd., Crosswell, UK) to the nearest 0.1 m, is measured standing upright, barefoot and in light clothing. The BMI is calculated as weight/height^2^ (kg/m^2^). Appendicular lean mass (ALM) is assessed by dual-energy x-ray absorptiometry (Horizon A scanner, Hologic Inc., Bedford, MA, USA). Low muscle mass is defined by the skeletal muscle index (SMI), which is the ALM (kg) divided by height^2^ (m^2^). A cut-off of SMI <  7.00 kg/m^2^ for men and SMI <  5.50 kg/m^2^ for women is used, according to the definition of the EWGSOP2 [3]. Handgrip strength was measured by hand dynamometry (Jamar 1, TEC Inc., Clifton, NJ, USA) whilst the participant is seated with the elbow at a 90 degree angle according the American Society of Hand Therapists [21] and according to the Southampton protocol, proposed by the EWGSOP2, hand grip strength is measured six times at alternating sides and the maximal value is reported [22]. The applied cut-off values are independent of BMI; low grip strength was defined as < 16 kg for women and <  27 kg for men [3]. Gait speed is assessed over a distance of six meters. Participants are instructed to walk 6 m on usual pace (walking aids allowed). The time is measured over four meters: from the moment the participant’s foot passes the mark of the one meter line on the ground until the foot passed the mark of the five meter line according to the BC guidelines [23]. The mean of three tests is calculated for scoring purposes. A cut-off of ≤0.8 m/s is used to define low gait speed [3]. The chair stand test is performed by measuring the time required to rise five consecutive times from a chair as quickly as possible with the arms folded in front of the chest [24]. The cut-off point for low physical performance is > 15 s, as defined according to the EWGSOP2 [3] (Table 1).

**Table 1:** EWGSOP1 and EWGSP2 diagnostic sarcopenia criteria

		Muscle strength			Muscle mass	Physical performance
		Handgrip strength		Chair stand	DXA	Gait speed
EWGSOP1 definition, used before April 2019	Men	BMI	HGS		< 7.26 kg/m^2^	≤ 0.8 m/s
		≤ 24	≤ 29			
		24.1–26	≤ 30			
		26.1–28	≤ 30			
		> 28	≤32			
	Women	BMI	HGS		< 5.45 kg/m^2^	
		≤ 23	≤ 17			
		23.1–26	≤ 17.3			
		26.1–29	≤ 18			
		> 29	≤ 21			
		≤ 23	≤ 17			
EWGSOP2 definition, used after April 2019	Men	< 27 kg		> 15 s for 5 rises	< 7.00 kg/m^2^	≤ 0.8 m/s
	Women	< 16 kg			< 5.50 kg/m^2^	

*EWGSOP* European working group on sarcopenia in older people, *DXA* dual-energy x-ray absorptiometry, *BMI* Body mass indexl, *HGS* handgrip strength, *s* second, *m* meter, *kg* kilogram

At screening a fasted blood sampling is checked for kidney function (inclusion if glomerular filtration rate > 30 ml/min/1.73m^2^), 25-hydroxyvitamin D and fasting glucose in order to comply with inclusion criteria. In case of low 25-hydroxyvitamin D blood concentration (< 20 ng/ml), participants are rescreened after 4 weeks supplementation with cholecalciferol (25.000 IU, once/week). After a positive evaluation of all inclusion criteria, participants are asked to complete a food diary to calculate the dietary protein intake. The flowchart of the study is presented in Fig. 1**.**

### Protocol amendment

A protocol amendment as from April 2019 resulted in an update of the inclusion criteria. This study protocol reports the inclusion criteria as from April 2019 and thus the inclusion of older people with probable, confirmed or severe sarcopenia according to the operational European definition of sarcopenia of the European Working Group on Sarcopenia in Older People (EWGSOP2) [3]. Before April 2019, presarcopenic or sarcopenic older people were included according to the operational definition of the EWGSOP1 [1]. From the participants with sarcopenia according to EWGSOP1, only participants that are also sarcopenic according to EWGSOP2 will be included in the analysis. EWGSOP1 differed with EWGSOP2 on following criteria. Low muscle mass was defined using a cut-off of SMI <  7.26 kg/m^2^ for men and SMI <  5.45 kg/m^2^ for women [1]. Handgrip strength was defined as the average value of three consecutive measurements of the dominant hand (according to Fried et al [25]) and the applied cut-off values are dependent of the BMI [1]. An overview of the different measures is reported in Table 1.

### Assignment of interventions and blinding

After receiving a written informed consent, the participants fulfilling the inclusion criteria are block randomized, stratified by sex, by means of a sealed envelope by an independent researcher. An R-script was developed to randomly allocate participants in one of five arms with a block size of (a multiple of) five. Randomly mixed block sizes are used and the block size is hidden from the study coordinators. Powder (protein and placebo) is packed in blank buckets, capsules (omega-3 fatty acids and placebo) are packed in blank pill boxes. Both participants and researchers are blinded to the protein and omega-3 fatty acids interventions, but not to the exercise intervention. The statistician is blinded for all treatment interventions. Unblinding of interventions during the trial is on researcher’s responsibility. For example, if the participant, general practitioner or researcher suspects an allergic reaction from one of the study products, unblinding the interventions is needed and the participant can no longer participate in the trial. An unblinded researcher is responsible for providing the powder (protein and placebo) and capsules (omega-3 fatty acids and placebo) to the blinded researchers, according to the participant’s allocated arm. This unblinded researcher does not participate in any of the study visits.

### Patient and public involvement

Preferences of older people for exercise and nutritional interventions were assessed and taken into account in the design of this study, as far as possible given the current state of the art [26].

### Interventions

#### Standard intervention

All participants complete a standard protocol. This standard protocol includes taking a vitamin D supplement, completing a trial diary, having a nutritional assessment, and wearing a monitor measuring movement. Placebo/control interventions for these standard interventions/assessments are not needed.

An oral vitamin D supplement (800 IU cholecalciferol, Vista D3–800, Vista-Life®) is taken daily from 28 days before the start of the intervention (preparation period), until the last day of the follow-up period. Compliance to vitamin D intake is measured by pill count of empty, full and open boxes.

Participants are asked to complete a four-day food diary to assess dietary energy and protein intake in the first week after the screening visit and at baseline visit and 3, 11 and 23 weeks. Participants record dietary intake in pre-defined household units on non-consecutive days including three week days and one weekend day. Dietary intake is computed based on the Belgian and USA Food Composition Table. The estimate of mean protein (g/kg BW/day, g/day or energy%/day) and energy (kcal/kg BW/day or kcal/day) consumption is calculated as a mean of four days. The personalized supplementation is calculated to reach 1.5 g protein intake/kg BW/day. Simultaneously, the Mini Nutritional Assessment - Short Form (MNA-SF), is used to identify older adults who are malnourished or at risk of malnutrition [27].

A trial diary is completed by all the participants during the intervention to record falls and intake of protein/omega-3 fatty acids/placebo supplements. During the contact moments in the follow-up period, the participants is motivated to complete the diary.

All participants wear an inertial measurement unit (IMU) (MoveMonitor +, McRoberts) to assess physical activities five days prior to the start of the study and the two first (Baseline-Week(W)2) and the two last weeks of the intervention (W10–12) and follow-up (W22–24). Participants allocated to the exercise intervention wear the IMU during the complete intervention period of twelve weeks to monitor compliance with the exercise program.

#### Exercise intervention

The exercise intervention in groups 1, 3 and 4 is the modified Otago Exercise Program (OEP). The OEP is a well-established home-based exercise program to prevent falls by improving muscle strength, balance and endurance in community-dwelling older persons [28]. Originally, it consists of warming-up, strengthening and balance retraining exercises and stretching, tailored to individual capabilities, complemented by a walking plan [28–30]. The modified OEP is a slightly altered version of the OEP to be in accordance with the recent guidelines of the American College of Sports Medicine [31], as suggested by later life training (Later Life Training Ltd., Amble, UK, (https://www.laterlifetraining.co.uk/llt-home-exercise-booklets/ ). More specifically, marching warming up exercises and an abdominal strength exercise were added, whereas stretching at the beginning of the program was omitted. The OEP exercises are progressive and the level of exercises is determined based on the physical capacity of each individual. Therefore, an individualized approach for each participant is possible.

One exercise session is organized prior to the start of the study. During this visit, the investigator explains all the exercises and determines the estimated 1-RM (repetition maximum) by the use of an ankle cuff weight. Based on this estimated 1-RM, the investigator determines the weight of the ankle cuff used during the study. The participants record in a dairy the weight of the ankle cuffs that were used at home to perform the exercises during the study period. During the first week, exercises are performed at 30% of 1-RM During each visit, the weights the participants used to exercise at home are discussed and the exercise performance is checked. When the exercises are performed correctly, participants start exercising at 60% of 1-RM. During the visits, the researcher prescribes, the weight of the ankle cuff in order to increase the intensity gradually increases up to 90% of 1-RM, after which a new 1-RM measurement takes place. Following new 1-RM measurements, the intensities increase again weekly from 60 to 90% of 1-RM. Participants are encouraged to perform the exercises three times a week and to walk at least 30 min twice a week, which may be split in three 10-min walks throughout the day.

Balance exercises are individualized with increasing difficulty levels, according to the participant’s progression. Four categories of exercises (with in total 9 levels) are developed for continuous incremental progression where the number and complexity of tasks increases, for example by reducing support, visual input or asking multi-tasking. Performance checks and individual intensity adjustments are performed at 1, 2, 4, 6, 8 and 10 weeks, based on progression on the score of the MiniBESTest [32]. Details of strength and balance progressive levels can be received upon request to the researchers.

No exercise intervention is prescribed for the participants in groups 2 and 5. Minimum 6 days and maximum 28 days before start of the intervention, the OEP is explained and practiced in groups of maximum four participants. Participants are provided with exercise booklets and ankle cuff weights. During the follow-up period the participants of the exercise intervention groups may continue the OEP, but no personal encouragement by the researchers is given during this period. No placebo intervention is foreseen for participants that do not receive an exercise intervention, participants without exercise intervention are asked to maintain their usual habits [33]. On participant’s request or on assessment of the researcher, an adapted exercise schedule can be assigned to the participant in exceptional cases e.g., pain during exercises. All researchers involved in visits regarding the OEP are certified as Otago Exercise Program leaders [34].

#### Nutritional intervention: protein supplementation

Participants in groups 2, 3 and 4 receive an individually adapted protein supplement to achieve a total (diet and supplements) daily protein intake of 1.5 g/kg BW [12] based on protein and energy intake calculated from a four-day food diary completed in the first week after the screening visit or subsequent food diaries. The 4-day estimated dietary records (EDR) is completed on non-consecutive days including three week- and one weekend day and during alternating periods to obtain a better estimate of the day to day variability [35]. Based on the formula of Beaton [36] including the within-subject standard deviation of older (65–85 years) adults living in the north of England [37], a 4-day EDR is appropriate to assess protein intake of an individual. At screening, W3 and W23, recorded days are Monday, Wednesday, Friday, Sunday and at baseline visit and W11, recorded days are Tuesday, Thursday, Saturday and Monday. Participants are instructed to meticulously record food and beverage intake for 24 h. Portion sizes are documented in pre-defined household units and the quality of the records is assured by reviewing and completing the records together with the participant. Dietary intake is computed based on the Belgian and USA Food Composition Table (Nubel, BE [38] and USDA FoodData Central, USA) [39]). The estimate of mean protein (g/kg BW/day or g/day) and energy (kcal/kg BW/day or kcal/meal/day) consumption is calculated as a mean of four days using the actual, uncorrected for BMI, body weight of the participant. Snacks are defined as the eating moments between meals. Snack 1 is defined as snacks between breakfast and lunch, snack 2 between lunch and dinner and snack 3 after dinner, before bedtime.

A meal is supplemented to achieve a combined (diet + supplement) protein intake of at least 25 g protein/meal (breakfast/lunch/dinner). However, if 1.5 g protein/kg BW/d is not yet reached by supplementing the meals to 25 g protein/meal, the combined (diet + supplement) protein intake is evenly increased over the meals (breakfast/lunch/dinner) up to a maximum of 35 g protein/meal and a maximum of 700 kcal/meal [40]. When, by doing this, 1.5 g protein/ kg BW/d is still not reached, additional protein supplement is given before bedtime (snack 3). The participants receive instructions on how to dissolve the protein powder and a range of suggestions of drinks and foods to mix with the powder before consumption. For that purpose, each participant receives an individualized drinking glass for each meal or snack that had to be supplemented. This procedure is completed for EDR at baseline (participant receives adapted drinking glasses at W2) and at W3 (participant receives adapted drinking glasses at W6). Calculations take into account the actual or ideal body weight of the participants. The actual body weight is used if this actual body weight complies with a BMI situated between 22 and 27 kg/m^2^. If the actual body weight results in a BMI higher than 27 kg/m^2^, the body weight is corrected to result in a corrected BMI of 27 kg/m^2^. Similarly, in case the BMI is below 22 kg/m^2^, the body weight is corrected to result in a corrected BMI of 22 kg/m^2^.

The protein powder contains 4.5 g protein/5 g powder (Resource® Instant Protein, Nestlé®). Participants in groups 1 and 5 receive an isocaloric placebo supplement (Resource dextrin maltose, Nestlé®). All supplements are manufactured by Nestlé Health Science and the nutritional contents of the protein and placebo supplement are shown in Table 2. The participant takes the protein supplement from 5 days before the start of the intervention. No protein supplement is taken during the follow-up period. Participants that also have an exercise intervention are asked to perform the OEP in close temporal proximity to one of the moments of the intake of the protein supplement.

**Table 2:** Nutritional content of the supplements

	Protein supplement 100 g	Isocaloric placebo 100 g
**Energy, kcal**	371	381
**Proteins, g (of which leucine, %)**	90 (9.71%)	0.2 (0%)
**Carbohydrates, g**	0.50	95
**Fat, g**	1.0	0.0

All supplements are manufactured and provided by Nestlé Health Science

#### Nutritional intervention: omega-3 fatty acids supplementation

Participants of group 4 receive commercially available omega-3 fatty acids to take with breakfast (Vista-Omega-3, Vista-Life®: 1 capsule providing 540 mg eicosapentaenoic acid (EPA) and 360 mg docosahexaenoic acid (DHA)), from one month before the start of the study until to end of intervention period. Groups 1, 2, 3 and 5 receive identical placebo capsules containing 1000 mg peanut oil.

### Outcome measurements

During the 12-week intervention period, study visits are planned at weeks 0 (baseline), 1, 2, 4, 6, 8, 10 and 12. During the 12-week follow-up period, there are 3 telephone contacts at weeks 16, 20 and 22 and one final visit at week 24. All assessments for outcome variables are performed at baseline, W12 and W24, except the hsCRP which is also measured at screening, W1 and W4. Visits in between baseline, W12 and W24 primarily aim consistent study proceeding, compliance measurement and adequate individualization and optimization of interventions. Study assessment procedures are described in Table 3.

**Table 3:** Study assessment procedures and timetable

	Enrollment	Allocation	Intervention period								Follow-up period			
Study week (W)	W-24 - W-4	W-4 - W0	Baseline	W1	W2	W4	W6	W8	W10	W12	W16^**a**^	W20 ^**a**^	W22^**a**^	W24
Inclusion criteria, informed consent	✔		✔							✔				✔
SF-36^**b**^ and SarQoL^**c**^ QoL measures, Short FES-I^**d**^, ADL^**e**^, cognitive test battery^**f**^, frailty^**g**^			✔							✔				✔
SPPB^**h**^, muscle strength (Biodex)^**i**^, muscle mass (BIA and DXA)^**j**^, BMI^**k**^			✔							✔				✔
Assess physical activity level^**l**^		✔	✔	✔	✔	✔	✔	✔	✔				✔	
Assess exercise compliance^**l,m**^ and MiniBESTest^n,m^			✔	✔	✔	✔	✔	✔	✔	✔				
Falls, use of health care			✔	✔		✔		✔		✔	✔	✔	✔	✔
MNA-SF^**o**^			✔				✔			✔				✔
Analyses food diaries		✔		✔		✔				✔				✔
(Dis) advantages of interventions assessment				✔		✔		✔		✔	✔	✔	✔	✔
Blood sample	✔	✔	✔	✔		✔	✔			✔				✔
Urine collection (24 h)			✔	✔	✔	✔	✔	✔	✔	✔				✔
Muscle sample			✔							✔				

^a^: telephonic visit; ^b^: SF-36: health related quality of life scale questionnaire [41]; ^c^: SarQol questionnaire [42]; ^d^: Short FES-I: falls efficacy scale international [43]; ^e^: ADL: activities of daily living according to Barthel index [44]; ^f^: cognitive test battery consisting of Repeatable Battery for the Assessment of Neuropsychological Status (RBANS) [45], Trail-Making Test, parts A and B [46], Maze test and Stroop test [47]; ^g^: frailty defined by Fried; ^h^: SPPB: short physical performance battery [25]; ^i^: muscle strength by biodex; ^j^: muscle mass by bioelectrical impedance analysis (BIA) and dual-energy x-ray absorptiometry (DXA); ^k^: BMI: body mass index; ^l^: compliance and physical activity measured by movement monitor; ^m^: only participants randomized into exercise group; ^n^: MiniBESTest [32]; ^o^: MNA-SF: mini nutritional assessment short form [27]

#### Primary outcome measure

The primary outcome measure of the RCT is the 12 week and 24 week change in Short Physical Performance Battery (SPPB) score [24] between group 1 (Ex) and group 3 (Ex + Prot). The SPPB includes three tests that assess static balance, gait speed and lower limb strength. Each test is scored from 0 to 4, and the total score is 0–12 points [41]. A higher score indicates a higher functional status.

#### Secondary outcome measures

The secondary outcomes are listed in Table 4.

**Table 4:** Secondary outcomes

Secondary outcomes	Baseline versus W12 change	W12 versus W24 change	Other time points
Muscle mass	X	X	
Muscle strength: knee and handgrip	X	X	
Body composition	X	X	
Balance	X	X	W1, W2, W4, W6, W8 and W10 in participants with exercise intervention to follow progression
Frailty	X	X	
Sarcopenia	X	X	
Activities of daily living	X	X	
Physical behaviour	X	X	
Quality of life	X	X	
Number of falls and fear of falling	X	X	
Malnutrition	X	X	W6
Cognitive functioning	X	X	
Dietary intake	X	X	W1, W3, W11
Use of health care	X	X	W1, W4, W8, W16, W20, W22,
Benefits and adverse events			W1, W4, W8, W16, W20, W22, W24
Blood measures	X	X	Vit D: screening
Subjective compliance with the interventions and vitamin D intake	X		W1, W2, W4, W6, W8 and W10
Objective compliance with the interventions	X		
Muscle parameters	X		

##### Clinical outcomes

In an exploratory way, the different effects of intervention arms; group 1 (Ex) and group 2 (Prot) compared to group 5 (Ctr), group 3 (Ex + Prot) compared to group 4 (Ex + Prot + omega-3 fatty acids) are assessed. Muscle mass (ALM) is measured by whole-body DXA scans. Muscle strength is assessed in two ways. Knee-extensor and knee-flexor strength of dominant leg are tested after standardized warm-up whilst positioned in equipment with arms resting on legs, and trunk and hip strap stabilized. Isometric (60° and 90°), isotonic (40, 20, 1 and 60% of the individual isometric 1-RM) and isokinetic (60°/s and 180°/s) (Biodex System 3 Pro Multijoint System isokinetic dynamometer) strength is measured as in Baggen et al. [42] with two minute rest period between testing conditions. Hand grip strength (Jamar 1 hand-held dynamometer) is measured as described above. Body composition is assessed by bioelectrical impedance analysis (BIA) using Tanita TBF-300, Tanita Cooperation, Tokyo, Japan; Omron BF-300, Omron Healthcare Co, Ltd., Kyoto, Japan; Bodystat 1500, Bodystat, Isle of Man, UK; and Bodystat Quadscan 4000, Bodystat, Isle of Man, UK. Balance is assessed by Mini-BESTest [32]. Physical frailty stage is defined by Fried et al. (frail (3–5/5), prefrail (1–2/5), robust (0/5)) [25] and sarcopenia according to the EWGSOP1 and EWGSOP2 definitions. Activities of daily living (ADL) is assessed by Barthel-index [43]. Physical behaviour (PB) is measured by the MoveMonitor MM(+) [44]. Health-related quality of life is assessed in two ways, the SF-36 questionnaire [45] and the SarQol questionnaire [46]. The number of falls is recorded and the circumstances, identified using weekly fall calendars and the fear of falling is assessed by falls efficacy scale international (Short FES-I) [47]. Malnutrition is assessed by the (MNA-SF [27]), and the dietary intake (protein and energy intake) by the four-day food diary. Cognitive functioning is measured by Repeatable Battery for the Assessment of Neuropsychological Status (RBANS) (immediate and delayed memory, attention, language and visuospatial skills [48]); Trail-Making Test, parts A and B (executive function/mental flexibility [49]); Maze test (planning capacity and foresight [50]); Stroop test (interference [51]). Also the use of health care and the participant reported benefits and adverse events are recorded.

##### Blood measures

Hemoglobin, kidney function, serum albumin, fasting glucose, cholesterol (HDL, LDL, total), triglycerides, insulin, Insulin Growth Factor 1 (IGF-1), 25-hydroxyvitamin D, vitamin B12, hs-CRP (also assessed at W1 and W4), Interleukin-6 (IL-6), Interleukin-4 (IL-4), Interleukin-13 (IL-13), Interleukin-1 bèta (IL-1β), Tumor necrosis factor alpha (TNF-α), creatine kinase (by ELISA) and compounds in blood related to sarcopenia such as myostatin and activin A.

##### Compliance measures

Compliance of a participant to an intervention (%) is defined as the number of completed intervention sessions/intakes to all sessions/intakes per participant. Compliance to an intervention (%) is defined as the range of compliance (%) of all participants.

- All measures of compliance to the Otago exercise program (OEP) are assessed both objectively (by the Dynaport MoveMonitor+) and subjectively (as reported in diary), during the complete study period
  - The number of Otago sessions the participant performed/the number of Otago sessions the participants needed to perform. Accordingly, this is calculated for the walking program, strength and balance part of OEP and for the integral exercise intervention.
  - Length of exercise session (time)/expected length of exercise time
- Compliance with protein supplementation.
  - Objectively: change in nitrogen (N) content in 8 24 h urine samples by the Dumas method [52] to estimate rise in protein intake and Creatinine index to estimate the completion of the urine samples [53, 54]
  - Subjectively: as reported in diary: the number of nutritional intakes the participant took/the number of intakes of nutritional supplement the participant was prescribed, assessed at W1, W2, W4, W6, W8 and W10
  - Subjectively: Count and weight of returned powder boxes, assessed at W1, W2, W4, W6, W8 and W10
- Compliance with omega-3 fatty acids supplement
  - Objectively: analysis of change in RBC membrane fatty acid profile at allocation visit, baseline, W12 and W24 [55, 56].
  - Subjectively: as reported in diary: the number of nutritional intakes the participant took/the number of intakes of nutritional supplement the participant was prescribed, assessed at W1, W2, W4, W6, W8 and W10
  - Subjectively: Count and weight of returned capsules, assessed at W1, W2, W4, W6, W8 and W10
- Compliance with Vitamin D supplement, assessed by counting vitamin D boxes and pills, measured at each study visit

##### Muscle outcomes

A muscle biopsy from the *m. vastus lateralis* are taken whilst using a 5-mm Bergström-type needle. The biopsy is performed by a trained physician at the non-dominant side in fasted state. The procedure is executed according to the modified-Bergström technique using local anesthesia (2% Lidocaine hydrochloride solution, 10 mL) [57].

- Change in markers of muscle wasting (Ubiquitin-proteasome pathway: MURF1, Atrogin1, FOXO3; apoptosis: cytochrome c, p53, caspases) and regeneration (PAX7, MYF5, MyoD, Ki67, mTOR, AMPK) by western blotting or rt-PCR
- Changes in muscle histology:
  - regenerating fibers (hematoxyline & eosine staining to express the relative number of fibers with a central nucleus)
  - muscle fiber typing (immunohistochemical staining for myosin heavy chain isoforms)
  - myogenic precursors (Pax7, Myogenin, MyoD)
  - myosteatosis (Oil Red O staining with density-based quantification in Image J software)

### Statistical analysis

#### Sample size

With a sample size of *n* = 180, the RCT has adequate power (0.80 with a two-tailed alpha level of 0.05) to detect a time-averaged difference in SPPB scores between two treatment groups after intervention. A change of 1 point in SPPB score is a substantial meaningful change [58]. With a standard deviation of 1.5, two post-treatment outcome measurements and an assumed correlation of 0.9 between repeated measurements, the required sample size is 27 participants per group based on Diggle [59]. With a dropout rate of 25% (which is similar to other RCTs with sarcopenic older adults), 36 participants per group are needed. Blood samples (screening, allocation visit, baseline, W12, W24) are obtained from all participants (*n* = 180). Muscle samples are obtained (baseline, W12) from eight persons in each of the five intervention groups (*n* = 40), which is conform the state of the art about the number of muscle biopsies in clinical trials [60].

#### Statistical analyses

Data analysis of the RCT will be performed by the intention-to-treat principle. The primary analysis is the time-averaged difference in SPPB scores between treatment groups 1 (Ex) and 3 (Ex + Prot) after intervention (W12) and follow-up (W24). Data will be analyzed using linear mixed models with SPPB scores as response variable and group, time point, sex and baseline SPPB score as covariates. The analysis will be performed at the 5% significance level.

Secondary analyses are pairwise comparisons between the other treatment groups for the time-averaged difference in SPPB scores after intervention and after follow-up. We will compare group 1 (Ex) with group 5 (Ctr) to assess the effects of exercise, group 2 (Prot) with group 5 (Ctr) to assess the effects of proteins. We will compare group 3 (Ex + Prot) with group 4 (Ex + Prot + omega-3 fatty acids) to explore the additional effects of omega-3 fatty acids. Not only SPPB scores will be analyzed as response variable, but also other variables as mentioned in the section ‘outcomes’ will be compared between different groups, such as the effects on markers of muscle wasting, regeneration and metabolism. These exploratory analyses will be performed using linear mixed models. Group, time point, sex and baseline ‘variable’ score are included as covariates. Given the exploratory nature of the study, no correction for multiplicity will be performed. This exploratory part of the study may guide future research to focus on the most interesting (combination of) intervention(s).

### Ethics and dissemination

This study (S60763) has been approved by the Ethics Committee of University Hospitals Leuven (UZ Leuven) and is registered on ClinicalTrials.gov (NCT03649698). Written informed consent is obtained from each participant before any study procedure, which is performed according to good clinical practice [56]. This study protocol gives a detailed overview of the methodology of the ENHANce project based on the Standard Protocol Items: Recommendations for Interventional Trials (SPIRIT) guidelines [61].

Dissemination of the results will be through articles in scientific and professional journals both in English and in Dutch and by (inter) national conference proceedings. Adverse events are reported by the researchers. Data is monitored by the study coordinators and reported to principal investigator. All data is handled confidential and data access is limited to study coordinators. Protocol amendments are reported on ClinicalTrails.gov.

## Discussion

The ENHANce RCT will assess the physical and biochemical effects and the mechanisms of action of a combined individualized and optimized nutritional and exercise intervention in sarcopenic older adults. Besides the advantages of a prospective blinded RCT design, this trial also distinguishes itself by 1) the personalized and optimized intervention design, 2) the objective compliance measurements, 3) the supplementation of omega-3 fatty acids on top of combined anabolic interventions, 4) the fundamental muscle biopsy study.

The key strength of this study is the well-considered design. The design was developed taking into account the preferences of older adults regarding exercise interventions [26]. Since research shows that older adults prefer to exercise at home [62–64], an exercise program specifically designed to be performed at home is used. The OEP is a well-established and effective home-based exercise program aimed at preventing falls in older persons by improving muscle strength, balance and endurance. It consists of individually tailored progressive strengthening and balance retraining exercises, next to a walking plan [28, 65]. Slight adaptations to the OEP ensure that it meets the recommendations of the American College of Sports Medicine position stand of exercise and physical activity for older persons in terms of frequency, intensity and duration [66]. For example, the exercises are performed with at least 60% of 1-RM by adaptable ankle cuff weights to obtain muscle hypertrophy [11]. Also, a home-based exercise intervention is a non-invasive and inexpensive option which may be the most suitable for sarcopenic older adults who often have mobility problems. This is in contrast to most RCTs, where the training is performed in group sessions under the supervision of an instructor outside the home environment [10].

A unique characteristic of this trial is the focus on measuring compliance in an objective way. A recurring concern in other RCT’s in this area is that the compliance of the participants to the intervention was not measured, making it difficult to estimate the dose-response effect of each intervention. In that regard, this study aims to assess the compliance with the interventions in an objective way. Compliance with protein intake is measured by measuring the nitrogen in urine samples [52–54]. Compliance with omega-3 fatty acids intake is assessed objectively according to the change in RBC membrane fatty acid profile [55, 56]. Exercise intervention compliance is assessed by a monitor with advanced machine learning techniques. Finally, since both exercise interventions and participation in a study may lead to an increased level of physical activity (PA) such as longer daily walks, this study also aims to measure PA by the MoveMonitor. This will lead to the possibility to distinguish whether the effects on the outcome of an RCT, such as change in muscle strength, are affected by of the level of PA.

Next to a large set of serum biomarkers, this project also includes a muscle biopsy study in order to gain more insights in the molecular mechanism behind sarcopenia and the effects of anabolic interventions on sarcopenia. This technique has proven to be feasible in sarcopenic older adults, but data on the molecular effects of combined anabolic interventions remain scarce [67].

Some possible limitations of this study can be addressed. First, the sample size of this study is based on power calculations for the primary outcome, a decrease in physical performance, whilst secondary outcomes will be analyzed exploratory. This may be seen as a limitation, however, this might still provide valuable insights into the most promising potential biomarkers in muscle and blood samples, and in turn may be informative for further research. Second, an amendment of the study protocol seemed necessary. First inclusions, from February 2018, were based on the EWGSOP1 definition. Since an updated definition (EWGSOP2) was published in October 2018, the inclusion criteria for the RCT have been revised to bring them in line with current scientific evidence. Lastly, the interventions are optimized according to the state-of-the-art literature at the moment of trial design. However, the progress of the state-of-the-art revealed new insights in optimization of interventions, e.g., the benefits of protein intake before bedtime to overcome the long fasting period between dinner and lunch [68], which is not taken into account in this study. Sub-analyses of participants that received a nighttime snack, based on their protein intake, may be an option. Also, the individualization of the intervention is interpretable in different ways. For example, this study aims to optimize protein intake in accordance with guidelines specifically formulated for this population by expert groups [12, 69], based on the individual intake of the participant whilst the individual need is not assessed but assumed based on the literature. An example of adapting an intervention to the measured individual need of the participant is, instead of relying on recommendations formulated for specific population groups, the energy need could have been assessed by for example ventilated hood. In short, this study aims to assess the effects of interventions when optimized to an optimal level based on groups, instead of based on individual measurements.

This randomized controlled trial aims to obtain both clinical and fundamental data on the effects and the mechanism of action of a 12-week home-based exercise and nutritional intervention in sarcopenic older adults.

## Data Availability

Not applicable.

## Abbreviations

ADL: Activities of daily living
ALM: Appendicular Lean Mass
BIA: Bioelectrical impedance analysis
BMI: Body mass index
BW: Body weight
DHA: Docosahexaenoic acid
DXA: Dual-energy x-ray absorptiometry
ENHANce: Exercise and Nutrition for Healthy AgeiNg
EPA: Eicosapentaenoic acid
EWGSOP: European Working Group on Sarcopenia in Older People
FES-I: Falls efficacy scale international
G: Gram
IU: International Unit
Kg: kilogram
MMSE: Mini-Mental State Examination
MNA-SF: Mini Nutritional Assessment - Short Form
RBANS: Repeatable Battery for the Assessment of Neuropsychological Status
RCT: Randomized controlled trial
SPPB: Short physical performance battery
TMT: Trail-Making Test
QoL: Quality of Life
